# 

**Published:** 1900-02-03

**Authors:** 


**CADBURY'S COCOA** ABSOLUTELY PURE.

### THE HOSPITAL

**CADBURY'S COCOA** NO DRUGS OR ALKALIES USED.

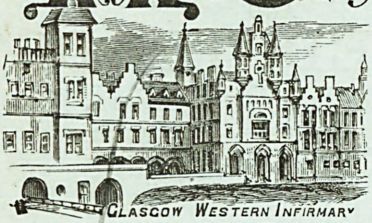

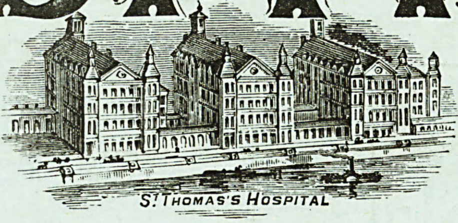

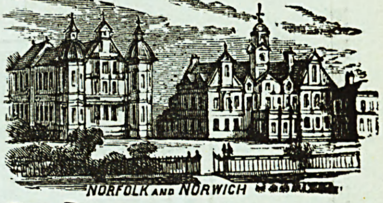

No. 697. Vol. XXVII. [Registered as a Newspaper.] SATURDAY, FEB. 3, 1900. [Subscription Terms, see p. 288.] **PRICE TWOPENCE.**

